# Effect of feeding pomegranate seed pulp on Awassi lambs’ nutrient digestibility, growth performance, and carcass quality

**DOI:** 10.14202/vetworld.2023.588-594

**Published:** 2023-03-22

**Authors:** Belal S. Obeidat

**Affiliations:** Department of Animal Production, Faculty of Agriculture, Jordan University of Science and Technology, Irbid 22110, Jordan

**Keywords:** Awassi lambs, carcass characteristics and meat quality, growth performance, pomegranate seed pulp

## Abstract

**Background and Aim:**

The use of alternative feeds in feeding livestock as an alternative to traditional feeds has been used for many years, on the one hand, to lower the price of feed and, on the other hand, to raise the profitability of raising livestock. The study aimed to investigate the effect of feeding pomegranate seed pulp (PSP) on the growth performance and carcass characteristics and the health of Awassi lambs.

**Materials and Methods:**

Twenty-four male lambs (16.9 ± 0.42 kg) were assigned randomly to one of two isonitrogenous (160 g/kg crude protein of dietary dry matter [DM]) treatment diets. The diets were the control (CON) and PSP-containing diet (100 g/kg of dietary DM; PSP100). The experimental period was 70 days preceded by 7 days of adaptation to diets. Feed intake was measured and lambs were weighed on day one and then biweekly. On day 49, eight lambs (four lambs per treatment) were chosen randomly and placed in metabolic cages for a digestibility trial. At the end of the trial, lambs were slaughtered to evaluate carcass characteristics and meat quality. The data were analyzed using Proc Mixed procedures of SAS.

**Results:**

The results revealed that nutrient intake was greater (p < 0.05) in lambs fed PSP100 than those fed the CON diet. Nitrogen intake and nitrogen retention were greater (p < 0.05) for lambs who consumed the PSP100 diet compared to CON. Final weight, total gain, and average daily gain were greater (p < 0.05) with lambs fed PSP100. Hot and cold carcass weights were higher (p < 0.05) by the PSP100 group than by the CON group. Carcass cut weight increased (p < 0.05) with feeding PSP100 diet. No differences were detected in blood parameters except high-density lipoprotein content, which was greater (p < 0.05) in the PSP100 group compared with the CON group.

**Conclusion:**

It could be concluded that adding PSP to lambs’ diets improved growth and carcass measurements positively and did not negatively affect lambs’ health; therefore, it is recommended to use PSP as an alternative to traditional feeds in lambs formulated rations.

## Introduction

Replacing traditional feedstuffs with alternative products enhanced sheep production in Jordan and worldwide [1]. Sheep farmers are facing many issues regarding increased feed costs, reduced production of traditional feeds, and decreased profitability. As a solution to these issues, replacing parts or complete kinds of traditional feeds with alternative sources was found to decrease production costs. It, therefore, improved productivity in the animal sector [2–4]. Plant-source by-products that are not edible for humans were being used as an alternative feed source for animals. Pomegranate by-products are an example of such feedstuff used recently and showed satisfying results regarding increasing ruminants’ productivity, reducing the cost of production, enhancing carcass characteristics and meat quality, and reducing feedstuff shortage and cost of waste disposal [5–7].

The pomegranate is considered as the oldest edible fruit [8, 9]. The pomegranate edible part makes up about 60% of the total fruit weight and contains about 75%–85% juice and 15%–25% seeds. Large quantities of residual are obtained from pomegranate industrial processing and form by-products such as seeds, peels, and pulp [10].

Pomegranate has numerous active phytogenic compounds, such as phenolic acids, tannins, and flavonoids. These many active components set behind considering pomegranate as an excellent alternative feed for improving growth performance, nutrient digestibility, and immunity [11]. Pomegranate by-products are produced by the extraction of pomegranate juice leaving proportions of seeds, peel, and residual pulp. Pomegranate by-products consist of sufficient amounts of crude protein (CP), fiber, and fat which make this product as an appropriate feed to be included within ruminants’ diets [12]. Therefore, the aim of this study was to investigate the effect of including pomegranate seed pulp (PSP) in on growth performance, carcass characteristics, and the health of Awassi lambs.

## Materials and Methods

### Ethical approval

The Jordan University of Science and Technology (JUST) Institutional Animal Care and Use Committee has given its approval to all of the procedures utilized in this study (#: 16/4/12/191).

### Study period and location

The study was conducted from July 2021 to October 2021 at the JUST Agricultural Research and Training Unit/Faculty of Agriculture. The samples were processed at Department of Animal Production Laboratory.

### Experimental design

From a group of 60 lambs who were born in Animal Farm at JUST, 24 heads of Awassi male lambs were randomly selected. Lambs were weighed, health assessed, and treated for internal parasites before the study started. No difference in initial body weight (BW) between the two groups was seen at enrolment; the average was 16.9 ± 0.42 kg. Lambs were kept separately in pens (1.5 × 0.75 m) that had a concrete floor. Each pen contained a plastic feeder and waterer (10 L). Throughout the experiment, lambs had unlimited access to water and feed (110% of the previous day’s intake).

Green Fields Oil Factory, Sweileh, Princess Haya Street No. 100, P.O. BOX 345, 11941 Al-Jubeiha, Amman, Jordan, donated PSP, which was delivered to the JUST campus, sun-dried, and ground before being added to the diet. A total of two isonitrogenous treatment diets (each containing 160 g/kg CP of dietary dry matter [DM]) were given to lambs (aged 2.5–3.5 months). Diets had been formulated to meet the needs of growing Awassi lambs[13]. To substitute some of the barley grain and soybean meal in the diets, either 0 g/kg PSP (control [CON]; n 12) or 100 g/kg PSP (PSP100) of dietary DM was used (Table-1). When the study was conducted, the price of each diet’s components on the local market was used to calculate the cost of each diet. The price of each diet included additional expenses, including labor, power, and water. The feed was mixed every 2 weeks during the trial, and a sample was taken to determine its chemical content. The experiment lasted for 70 days; the first 7 days were used to acclimate the animals to the diets and pens, and the remaining 63 days were used to gather data. Daily feed consumption was measured. At the start of the trial and then every 2 weeks after that, animals were weighed.

**Table-1:** Ingredients and chemical composition of diets-containing PSP fed to Awassi lambs.

Item	Diet^a^		

	CON	PSP100	PSP
Ingredients (g/kg DM)			
Barley grain, whole	475	515	
Soybean meal, 440 g/kg CP (solvent)	205	165	
PSP	0	100	
Wheat straw	300	200	
Salt	10	10	
Limestone	9	9	
Vitamin-mineral premix^b^	1	1	
Feed cost/ton (US$)^c^	404	362	
Nutrients (g/kg DM)			
Dry matter	908	910	976
Crude protein	160	161	168
Neutral detergent fiber	334	337	672
Acid detergent fiber	149	156	444
Ether extract	9.2	17.2	76.4

a Diets were: the CON diet or 100 g/kg PSP100 of dietary DM,

b Composition per kg contained (Vitamin A, 600,000 IU; Vitamin D3, 200,000 IU; Vitamin E, 75 mg, Vitamin K3, 200 mg; Vitamin B1, 100 mg; Vitamin B5, 500 mg; lysine 0.5%; DL-methionine, 0.15%; manganese oxide, 4000 mg; ferrous sulfate, 15,000 mg; zinc oxide, 7000; magnesium oxide, 4000 mg; potassium iodide, 80 mg; sodium selenite, 150 mg; copper sulfate, 100 mg; cobalt phosphate, 50 mg, dicalcium phosphate, 10,000 mg.

c Calculated based on the prices of diet ingredients of the year 2022. CON=Control, DM=Dry matter, PSP=Pomegranate seed pulp

Samples of diets and refusals were ground (Brabender OHG Duisdurg, Kulturstrasse 51–55, type 880845, Nr 958084, Germany) and stored for subsequent analysis to determine their chemical makeup. Using Association of Official Analytical Chemists methods [14], the ground samples were examined for DM, CP, and either extract (EE). Neutral detergent fiber (NDF) and acid detergent fiber (ADF) were examined in accordance with Van Soest *et al*. [15] protocol’s using the ANKOM2000 fiber analyzer apparatus (ANKOM Technology Cooperation, Fairport, NY, USA).

Four randomly chosen animals from each group were chosen on day 49 of the experiment and put in metabolic cages (1.05 0.80 m) to measure the nutrients’ ability to be digested and the N balance. Animals were given 5 days of adaptation to the metabolic cages before data collection began 5 days later (i.e., feed intake and refusals, fecal, and urine output). Then, 5% of the urine and 10% of the feces were stored at −20°C for DM, CP, NDF, ADF, and EE analyses. Nitrogen content in urine samples was examined (Kjeldahl procedure).

At 8:00 am at the beginning and at the end of the study, simple vacutainers were used to collect blood samples from the jugular vein (before feeding). After 1 h of collection, blood samples were centrifuged at 1734× *g* for 15 min. On the day of analysis, serum samples were promptly separated and kept at 20°C. All serum concentrations, including those for glucose, urea N, alanine aminotransferase (ALT), aspartate aminotransferase (AST), alkaline phosphatase (ALP), total protein, and albumin, were measured using a spectrophotometer (JENWAY 6105 UV/Vis, Model 6105, Jeneway LTD Felsted, Dunmow ESSEX CM6 3LB, UK) following the directions of commercial kits.

### Slaughtering procedure and meat quality measurements

All used animals were slaughtered at the Animal Farm facilities in the Agricultural Training and Research Unit at JUST at the conclusion of the study to assess carcass qualities. Lambs were slaughtered by trained individuals at 9:00 am after having fasted for 18 h. Fasting live weight and hot carcasses were measured right before and after slaughter, respectively. Immediately on slaughter, non-carcass edible parts (lungs and trachea, heart, liver, spleen, kidneys, renal fat, mesenteric fat, and testes) were removed and weighed.

To determine the dressing percentage (cold carcass weight/fasted live weight), the carcasses were chilled for 24 h at 4°C. Then, on the carcasses and longissimus muscle, linear measurements (tissue depth [*GR*], rib fat depth [*J*], eye muscle width [*A*], eye muscle depth [*B*], eye muscle area, and fat depth [*C*]) were measured. The carcasses were then divided into four pieces (shoulder, rack, loin, and leg cuts). After cutting, the longissimus muscle is removed from the loin cut, vacuum-packed right away, and kept at −20°C for 2 weeks to test the meat’s quality. Meat quality measurements on longissimus muscles after thawing in a chiller at 4°C included shear force values, color (CIE L*a*b* coordinates), water holding capacity, pH, and cooking loss.

### Statistical analysis

All data were analyzed using SAS’ PROC MIXED techniques, with the treatment diet serving as the only fixed effect (Version 8.1, 2000, SAS Inst. Inc., Cary, NC). Lambs were used as a random variable. The least-square means have been separated using the relevant pair-wise t-tests if the fixed effects were significant (p ≤ 0.05).

## Results

The chemical composition of PSP which is included in the experimental ration, is presented in Table-1. The data in this table revealed that PSP is rich in NDF, ADF, and EE. Data from this table cleared that the experimental rations (CON, and PSP100) were formulated as isonitrogenous with CP of 160 g/kg of dietary DM. Both rations were almost similar in NDF content (334 g/kg DM for CON and 337 g/kg DM for PSP100), while PSP100 had greater ADF and EE content than the CON. The cost of the PSP100 diet was lower than the cost of the CON diet.

Results from the effect of feeding PSP on nutrient intake, digestibility, nitrogen balance, and growth performance of Awassi lamb are shown in Table-2. The intake of all nutrients (DM, NDF, ADF, EE (g/d), and metabolizable energy, Mcal/kg) was greater (p < 0.05) for lambs who consumed the PSP100 diet. Feeding PSP did not affect the nutrients digestibility coefficient (p > 0.05). Nitrogen intake (g/d) and nitrogen retained (g/d) were greater (p < 0.05) for lambs consumed the PSP100 diet compared to CON, while feces and urine N (g/d) and N retention were not affected (p > 0.05) with the dietary treatments. The lambs fed the PSP diet had greater (p < 0.05) final weight (kg), total gain (kg), and average daily gain (ADG; g/d) compared to lambs fed the CON.

**Table-2:** Effects of feeding PSP on nutrient intakes, digestibility, N balance, and growth performance of Awassi lambs.

Item	Diet^a^			

	CON (n = 12)	PSP100 (n = 12)	SEM	p-value
Nutrient intake, g/d				
Dry matter, g/d	1033	1273	42.9	0.001
Crude protein, g/d	165	205	6.9	0.001
Neutral detergent fiber, g/d	345	429	14.4	0.001
Acid detergent fiber, g/d	154	199	6.7	0.002
Ether extract, g/d	10	22	0.7	<0.0001
Metabolizable energy, Mcal/kg	2.36	3.11	0.10	0.001
Digestibility coefficients				
Dry matter	77.58	80.37	1.742	0.339
Crude protein	73.69	77.39	2.30	0.270
Neutral detergent fiber	60.38	64.02	5.96	0.637
Acid detergent fiber	55.96	59.06	4.72	0.674
Ether extract	74.69	76.58	3.60	0.720
N balance				
N intake, g/d	22.71	25.47	0.417	0.019
N in feces, g/d	5.97	5.78	0.729	0.764
N in urine, g/d	2.92	3.29	0.247	0.272
N retained, g/d	13.83	16.41	0.752	0.043
Retention, g/100 g	60.85	64.55	3.315	0.157
Growth performance				
Initial weight, kg	16.42	15.83	0.265	0.111
Final weight, kg	29.9	31.77	0.321	0.001
Total gain, kg	13.5	15.9	0.37	<0.0001
Average daily gain, g/d	214	253	5.9	<0.0001
Feed efficiency (DMI: ADG)^b^	4.85	5.04	0.162	0.424

a Diets were: the CON diet or 100 g/kg PSP100 of dietary DM,

b DMI: ADG=Dry matter intake: average daily gain. CON=Control, DM=Dry matter, PSP=Pomegranate seed pulp, SEM=Standard error of the mean

Results of feeding Awassi lambs’ PSP and its effect on carcass and non-carcass components illustrated in Table-3 showed that fasting live weight (kg) was greater (p < 0.05) for lambs fed PSP100 compared to the CON. Hot carcass weight and cold carcass weight (kg) significantly differ and were higher (p < 0.05) for the PSP100 group than the CON group. The dressing percentage and non-carcass components were not affected (p > 0.05) by feeding the PSP diet. Carcass cut weight (kg) increased (p < 0.05), while fat-tail tended to be greater (p = 0.07) with the PSP100 group. Carcass leaner dimensions (Table-4) and meat quality characteristics (Table-5) did not differ (p > 0.05) by feeding PSP100 to Awassi lambs compared to CON-fed lambs.

**Table-3:** Effects of feeding PSP on carcass and non-carcass components of Awassi lambs.

Item	Diets^a^			

	CON (n = 12)	PSP100 (n = 12)	SE	p-value
Fasting live weight (kg)	28.8	31.6	0.57	0.006
Hot carcass weight (kg)	13.7	15.3	0.45	0.026
Cold carcass weight (kg)	13.1	14.6	0.41	0.029
Dressing percentage	45.45	46.03	0.712	0.575
Non-carcass components (kg)^b^	1.39	1.39	0.024	0.920
Carcass cut weights (kg)^c^	11.54	12.50	0.281	0.034
Fat-tail (kg)	1.41	1.68	0.101	0.072

a Diets were: the CON diet or 100 g/kg PSP100 of dietary DM,

b Non-carcass components (Heart, liver, spleen, kidney, and lungs and trachea),

c Carcass cut (shoulder, racks, loins, and legs). CON=Control, DM=Dry matter, PSP=Pomegranate seed pulp, SE=Standard error

**Table-4:** Effects of feeding PSP on carcass leaner dimensions of Awassi lambs.

Item	Diets^a^			

	CON (n = 12)	PSP (n = 12)	SE	p-value
Leg fat depth (*L3*) (mm)	2.19	2.45	0.182	0.195
Tissue depth (*GR*) (mm)	7.94	8.42	0.367	0.370
Rib fat depth (*J*) (mm)	1.72	1.85	0.1279	0.469
Eye muscle width (*A*) (mm)	49.71	48.90	0.533	0.138
Eye muscle depth (*B*) (mm)	19.47	19.92	0.240	0.218
Fat depth (*C*) (mm)	1.41	1.33	0.1317	0.666
Shoulder fat depth (*S2*) (mm)	1.14	1.14	0.088	0.964

a Diets were: the CON diet or 100 g/kg PSP100 of dietary DM. CON=Control, DM=Dry matter, PSP=Pomegranate seed pulp, SE=Standard error

**Table-5:** Effects of feeding PSP on meat quality characteristics of Awassi lambs.

Item	Diets^a^			

	CON (n = 12)	PSP100 (n = 12)	SE	p-value
pH^b^	5.73	5.8	0.01	0.124
Cooking loss (g/100 g)	39.3	39.0	0.37	0.478
Water holding capacity (g/100 g)	26.1	28.0	0.87	0.134
Shear force (kg/cm^2^)	8.2	9.0	0.39	0.195
Color coordinates				
L* (whiteness)	37.1	37.1	0.52	0.995
a* (redness)	27.68	26.44	0.204	0.557
b* (yellowness)	18.47	18.22	0.336	0.599

a Diets were: the CON diet or 100 g/kg PSP100 of dietary DM,

b pH measured after thawing. CON=Control, DM=Dry matter, PSP=Pomegranate seed pulp, SE=Standard error

Blood parameters are presented in Table-6. No differences (p > 0.05) were noticed in the blood metabolites when feeding PSP except for the high-density lipoprotein (HDL) content which was greater (p < 0.05) compared to CON. Blood urea N, blood glucose, cholesterol, triglycerides, low-density lipoprotein (LDL) (mg/dL), AST and ALT (IU/L), ALP (IU/L), and creatinine (mg/dL) content were the same among the different diets (p > 0.05).

**Table-6:** Effects of feeding PSP on blood parameters of Awassi lambs.

Item	Diets^a^			

	CON (n = 12)	PSP100 (n = 12)	SE	p-value
Urea N, mg/dL	17.7	19.5	1.30	0.318
Glucose, mg/dL	55.1	56.2	2.01	0.724
Cholesterol, mg/dL	41.3	48.0	2.68	0.107
Triglycerides, mg/dL	14.5	14.9	1.26	0.854
HDL, mg/dL	28.2^a^	32.8^b^	1.08	0.012
LDL, mg/dL	9.7	10.9	2.05	0.6506
AST, IU/L	60.3	52.4	6.5	0.405
ALT, IU/L	11.0	8.6	1.31	0.153
ALP, IU/L	85.6	85.5	6.19	0.981
Creatinine, mg/dL	0.62	0.70	0.077	0.515

a Diets were: the CON diet or 100 g/kg PSP100 of dietary DM. CON=Control, DM=Dry matter, PSP=Pomegranate seed pulp, SE=Standard error, HDL=high-density lipoprotein, LDL=Low-density lipoprotein, ALT=Alanine aminotransferase, AST=Aspartate aminotransferase, ALP=Alkaline phosphatase

## Discussion

The chemical composition of pomegranate by-products varied in fiber and ether extract contents as reported in the previous studies [6, 16]. The differences in these contents could be referred to a type of pomegranate processing, pomegranate varieties, and the type of by-product mixture [17]. In this present study, PSP was obtained after oil and juice extraction. Despite the extraction, the content of EE remained greater with the diet containing PSP compared to the CON diet, which was reflected in the higher energy content that was consumed by lambs offered the PSP100 diet. However, the other nutrients were approximately similar between the two diets.

In line with earlier research that looked at alternate feedstuffs [3, 4, 18], the cost of the PSP100 diet was lower than the CON diet by 10.4%. These results might be explained by the fact that alternative feeds and/or agro-industrial by-products are less expensive than traditional feeds. If using alternative feeds has no negative effects on the health and performance of the animals consuming them, it will lower the cost of feed, which will increase the profitability of owning these animals.

The improvement of feed intake by ruminants fed pomegranate by-products was reported previously [5, 19]. In this study, the addition of PSP to the diet increased lambs’ nutrient intake significantly. This enhancement of total feed intake possibly referred to the diet palatability shown with lambs fed the PSP diet compared to the CON; since feed selection of lambs depends mainly on the palatability of the feed [19]. Opposite to this result, including PSP did not affect animal’s feed intake as reported by others [20, 21]. The previous studies connected the adverse effect of including PSP in ruminants’ diets with the level of tannins found in pomegranate by-products [22]. Overall, the results of the intake assured that the use of PSP did not impact the nutrient intake indicating that this production would increase the profitability of the sheep raisers as well as mitigate environmental pollution.

Nutrient digestibility was not affected by the inclusion of PSP in this present study. On the contrary, previous studies reported that pomegranate by-products in addition to animals’ diets affected digestibility coefficient values [5, 23, 24]. Nitrogen balance, in this present study, was affected by PSP through improvement in nitrogen intake and retention. Utilization of nitrogen with ruminants consuming feeds containing tannins (such as pomegranate by-products) was reported to be improved [19]. Tannins protect dietary protein at rumen by reducing the microflora effect and with that increasing the rate of absorption of amino acids in the intestine [25].

Lambs’ performance improved by adding PSP to their diets. In this present study, the improvement in weight gain and ADG might be connected to the enhancement of nutrient intake of lambs throughout the experimental period. In agreement with this result, El-Elaime [24] reported that by increasing the amount of pomegranate by-products added to the experimental diets, lamb’s daily gain increased accordingly. Other studies likewise confirmed that adding pomegranate by-products would improve lamb’s final BW, weight gain, feed intake, and efficiency when compared to lambs fed the CON diets [6, 19, 22, 26], on the other hand, noticed that increasing the inclusion level of pomegranate seed cake more than 100 g/kg DM did not affect palatability and digestion, nor DM intake and growth rate by lambs consumed the different diets.

Finishing lambs on PSP-containing diet improved carcass quantitative traits. Increasing both hot and cold carcass weight, as well as, carcass cut weight might be explained by the improvement of weight gain during the experimental period. The results of this study were opposite to what was reported previously that reported that the inclusion of pomegranate by-products did not affect animals’ carcass characteristics [6, 23]. In this study, the fat-tail was slightly increased with the PSP group, while carcass leaner dimensions and meat quality were not affected. Kotsampasi *et al*. [20] noticed that as the PSP inclusion level increased, carcass qualitative traits improved. The authors referred to the improvement in the high content of antioxidants provided by pomegranate by-products. Moreover, meat color and lipid chemical forms could be affected by the content of the antioxidant compounds as noticed by Amaral *et al*. [27]. Hereof, the addition of pomegranate by-products might impact meat and carcass characteristics, depending on the inclusion level.

Feeding pomegranate by-products to lambs showed no significant effect on kidney functioning enzymes and blood metabolites, in addition to not revealing any adverse impact on their health as reported by others [7, 28]. The inclusion of pomegranate, in this present study, had no negative effects on blood metabolites. However, HDL content increased with lambs consumed a diet containing PSP. Zeweil *et al*. [29] reported similar results when noticing an increase in HDL and LDL/HDL ratio with rabbits who consumed the pomegranate-included diets. On the other hand, Khan *et al*. [30] stated that pomegranate addition to sheep’s diets did not affect serum HDL density. Pomegranate, as reported by Aviram and Rosenblat [31], has a superior ability to protect HDL (known as the good cholesterol) from oxidation, as well as increase the activity of associated HDL enzymes (such as paraoxonase 1; PON1), which act on breaking down oxidized lipids that might negatively affect lipoproteins and with that enhance individuals’ health. These results indicated that the inclusion of PSP at 100 g/kg of dietary DM did not affect the health of the lambs, and therefore, it can be recommended as a part of the ruminants’ diet.

## Conclusion

The inclusion of PSP as 100 g/kg DM resulted in lambs’ productive performance and carcass traits enhancement. Pomegranate seed pulp can be added into total mixed rations for growing Awassi lambs with no adverse effects on meat quality and animal health. Therefore, PSP can be considered a low-cost alternative feed ingredient for ruminants, whereas reducing waste disposal of pomegranate-related products manufacturing.

## Author’s Contributions

BSO: Designed and conducted the study and drafted, revised, and approved the final manuscript.
